# Vinorelbine/carboplatin *vs* gemcitabine/carboplatin in advanced NSCLC shows similar efficacy, but different impact of toxicity

**DOI:** 10.1038/sj.bjc.6603869

**Published:** 2007-06-26

**Authors:** N Helbekkmo, S H Sundstrøm, U Aasebø, P Fr Brunsvig, C von Plessen, H H Hjelde, O K Garpestad, A Bailey, R M Bremnes

**Affiliations:** 1 Institute of Clinical Medicine, University of Tromsø and Department of Oncology, University Hospital of Northern Norway, 9038 Tromsø, Norway; 2 Department of Oncology, St Olavs University Hospital, 7030 Trondheim, Norway; 3 Department of Pulmonology, University Hospital of Northern Norway and Institute of Clinical Medicine, University of Tromsø, 9038 Tromsø, Norway; 4 Department of Oncology, Rikshospitalet-Radiumhospitalet HF, Ullernch. 70, 0310 Oslo, Norway; 5 Department of Thoracic Medicine, Haukeland University Hospital and Institute of Medicine, University of Bergen, Jonas Lies vei, 5021 Bergen, Norway; 6 Department of Pulmonology, St Olavs University Hospital, 7030 Trondheim, Norway; 7 Thoracic Department, Division of Internal Medicine, Stavanger University Hospital, 4010 Stavanger, Norway; 8 Department of Pulmonology, University Hospital of Akershus, Sykehusv. 27, 1474 Nordbyhagen, Norway

**Keywords:** non-small-cell lung cancer, vinorelbine, gemcitabine, palliative, quality of life, survival

## Abstract

This randomised phase III study in advanced non-small cell lung cancer (NSCLC) patients was conducted to compare vinorelbine/carboplatin (VC) and gemcitabine/carboplatin (GC) regarding efficacy, health-related quality of life (HRQOL) and toxicity. Chemonaive patients with NSCLC stage IIIB/IV and WHO performance status 0–2 were eligible. No upper age limit was defined. Patients received vinorelbine 25 mg m^−2^ or gemcitabine 1000 mg m^−2^ on days 1 and 8 and carboplatin AUC4 on day 1 and three courses with 3-week cycles. HRQOL questionnaires were completed at baseline, before chemotherapy and every 8 weeks until 49 weeks. During 14 months, 432 patients were included (VC, *n*=218; GC, *n*=214). Median survival was 7.3 *vs* 6.4 months, 1-year survival 28 *vs* 30% and 2-year survival 7 *vs* 7% in the VC and GC arm, respectively (*P*=0.89). HRQOL, represented by global QOL, nausea/vomiting, dyspnoea and pain, showed no significant differences. More grade 3–4 anaemia (*P*<0.01), thrombocytopenia (*P*<0.01) and transfusions of blood (*P*<0.01) or platelets (*P*<0.01) were observed in the GC arm. There was more grade 3–4 leucopoenia (*P*<0.01) in the VC arm, but the rate of neutropenic infections was the same (*P*=0.87). In conclusion, overall survival and HRQOL are similar, while grade 3–4 toxicity requiring interventions are less frequent when VC is compared to GC in advanced NSCLC.

Lung cancer remains the leading cause of cancer death worldwide, and the incidence is increasing (Jemal *et al*, 2006). Non-small cell lung cancer (NSCLC) accounts for about 80% of lung cancer cases, and the majority presents with locally advanced or metastatic disease (Ries *et al*, 2006). The patient population is large, median age high and comorbidity often considerable. Optimising the treatment is a challenge. Any systemic anticancer therapy to this group should be effective, tolerable and improve quality of life (QOL).

In the meta-analysis from 1995 (Alberti, 1995) demonstrated superiority of chemotherapy over best supportive care in advanced NSCLC. Since then, new agents like vinorelbine, gemcitabine, taxans and irinotecan, often referred to as third-generation drugs, have established their role in this disease. The third-generation agents have been compared to older regimens in various ways. Platinum-based doublets with a third-generation drug have proven more effective than monotherapy, and equally effective, but less toxic, than three-drug regimens (Bunn, 2002). Two-drug combination regimens have been established as recommended first-line chemotherapy in advanced NSCLC (Pfister *et al*, 2004). Although slightly inferior to cisplatin (Hotta *et al*, 2004), carboplatin is advocated a valuable alternative in the palliative treatment of NSCLC. Which third-generation non-platinum agent to choose is, however, still debated.

Vinorelbine/carboplatin (VC) and gemcitabine/carboplatin (GC) are both third-generation combinations used in the treatment of NSCLC. Vinorelbine in combination with a platinum-compound is established as treatment of advanced NSCLC (Kelly *et al*, 2001; Scagliotti *et al*, 2002; Plessen *et al*, 2006). The gemcitabine/platinum combination is widely used and reported to be effective and tolerable (Le Chevalier *et al*, 2005). The aim of the present study was to compare VC and GC with respect to efficacy, health-related quality of life (HRQOL) and toxicity in stage IIIB/IV NSCLC patients. The inclusion criteria were liberal, reflecting the everyday clinical situation. To our knowledge, this is the first randomised direct comparison of these two regimens.

## PATIENTS AND METHODS

### Patients

In this national, multicentre and randomised phase III trial, chemonaive patients with histologically or cytologically confirmed NSCLC stage IIIB or IV, not candidates for curative treatment, were included. Eligibility criteria were WHO performance status (PS) 0–2 and ability to understand oral and written study information. No upper age limit was defined. White blood-cell count >3.0 × 10^9^ cells l^−1^, platelet count >100 × 10^9^ cells l^−1^, serum creatinin<1.5 times upper reference limit and bilirubin and serum transaminase levels<2 times upper limits were required. Exclusion criteria were other active malignancies, pregnancy, or breast feeding.

The Regional Ethical Review Board, the Norwegian Social Science Data Services and the Norwegian Medicines Agency have approved the study.

### Baseline investigation

At study entry, all patients underwent clinical examinations, laboratory measures, chest X-ray and chest CT scan including upper abdomen. PS, body weight and height were registered. Patients were staged according to the clinical stage classification from 1997 (Mountain, 1997).

### Randomisation

After signing the informed consent and completing the baseline HRQOL form, patients were randomised to receive VC or GC, stratifying for PS 0–1 *vs* 2 and stage IIIB *vs* IV. Randomisation was performed by phone or fax to the randomisation centre (Clinical Cancer Research Office, University Hospital of Northern Norway).

### Chemotherapy

In each arm, three courses of chemotherapy, primarily administered on an outpatient basis, were given at 3-week cycles. Carboplatin was administered on day 1, and vinorelbine or gemcitabine on days 1 and 8 in each course. For both regimens, the carboplatin dose was calculated by the Chatelut formula (Chatelut *et al*, 1995) using AUC=4, which approximates Calvert (Calvert *et al*, 1989) AUC=5.

Carboplatin in 500 ml 5% glucose was administered as a 1-h infusion. Vinorelbine 25 mg m^−2^ in 100 ml 5% glucose was given as a 10 min i.v. infusion, whereas gemcitabine 1000 mg m^−2^ in 250 ml 0.9% NaCl was administered i.v. for 30 min. Patients ⩾75 years received 75% of standard doses. Antiemetics were given before chemotherapy.

Blood counts were assessed weekly. If moderate haematological toxicity (WBC: 2.5−2.99 × 10^9^ l^−1^ and/or platelets: 75–99 × 10^9^ l^−1^ occurred at days 1 and 8, doses were reduced by 25%. In case of severe haematological toxicity (WBC<2.5 × 10^9^ l^−1^ and/or platelets< 75 × 10^9^ l^−1^), chemotherapy was postponed for 1 week and further doses were reduced by 25%. If treatment was associated with febrile leucopoenia or leucopoenia-associated infection, chemotherapy was postponed until clinical recovery and further doses were reduced by 25%. Treatment was discontinued in case of disease progression, unacceptable toxicity or on patient's request.

### Patient follow-up

At start of each treatment cycle (weeks 0, 3 and 6) and at the 8-weekly follow-up visits (weeks 9, 17, 25, 33, 41 and 49), patients underwent clinical examinations, evaluations of PS, assessments of body weight, laboratory tests and chest X-rays. For evaluation of disease progression, chest CT was performed when indicated.

Haematological toxicity was graded according to the WHO toxicity criteria (WHO, 1979). Transfusions, bleedings, leucopoenic infections, use of G-CSF or erythropoietin and hospital admissions due to treatment toxicity were registered. The use of radiotherapy or second-line chemotherapy was recorded.

Site visits were performed at hospitals, which included ⩾20 patients. Otherwise, missing data were retrieved through phone or mail to the patient's physician.

### Assessment of HRQOL

HRQOL was assessed using the European Organization for Research and Treatment of Cancer (EORTC) Quality of Life Questionnaire (QLQ)-C30 (Aaronson *et al*, 1993) and the lung cancer-specific module QLQ-LC13. (Bergman *et al*, 1994) The QLQ-C30 measures fundamental aspects of HRQOL and symptoms commonly reported by cancer patients in general, while the QLQ-LC13 addresses symptoms specifically associated with lung cancer and its treatment.

Baseline HRQOL questionnaires were completed before the first chemotherapy cycle. Follow-up questionnaires (before each cycle and every 8 weeks until 49 weeks) were mailed to the patients from the randomisation office. In lack of response, one reminder was mailed after 14 days.

### Study endpoints

The main endpoint was overall survival. Further endpoints were patient-assessed HRQOL and treatment toxicity including required interventions. Global QOL, nausea/vomiting, dyspnoea and pain during the first 17 weeks were pre-defined as the primary HRQOL items of interest.

Global QOL is an important general measure, nausea/vomiting a common side effect of chemotherapy and dyspnoea a severe symptom in lung cancer. Pain is frequent in stage IV disease with a significant impact on QOL.

### Statistical considerations

Estimation of study size was based both on survival and HRQOL measures. To detect a difference in survival of 11% or HRQOL of 15% between the groups, provided a power of 80% and a significance level of 0.05 using two-sided tests, 380 patients were required. Based on a 5% dropout, the required patient number was 400.

Survival, defined as time from randomisation to the date of death, was compared using Kaplan–Meier estimates and the log-rank test.

HRQOL items for global QOL, nausea/vomiting, dyspnoea and pain were scored for each patient according to the EORTC QLQ-C30 scoring manual (Fayers *et al*, 2001). All HRQOL item scores range from 0 to 100. A high score in global QOL represents a good QOL, whereas a high symptom-scale score represents more symptoms. For group comparisons of baseline scores during and after chemotherapy, and changes in scores from baseline, the Mann–Whitney *U*-test was used. A mean change of ⩾10 was considered clinically relevant and significant (Osoba *et al*, 1998). The AUC from baseline to week 17 was compared using a two-sided *t*-test. Group differences consistent across all three methods of analysis, or yielding a *P*-value ⩽0.01, were considered significant.

Differences in haematological toxicity and registered interventions were analysed using two-sided *t*-test and *χ*^2^ tests.

## RESULTS

### Patients

**Figure 1:**
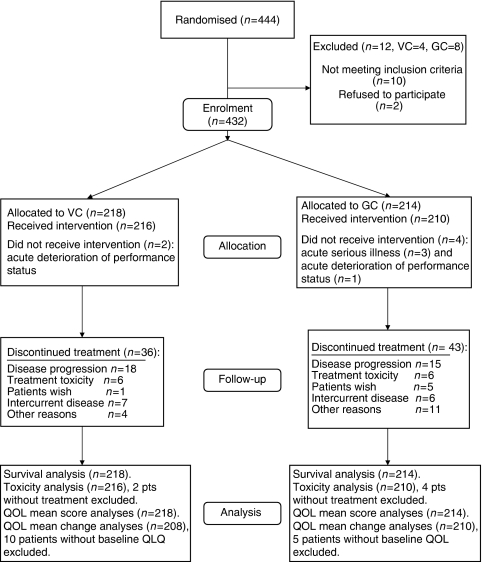
Flow of patients through each stage of the study.

Between October 2003 and December 2004, 444 patients from 33 hospitals in Norway were randomised to receive VC (*n*=222) or GC (*n*=222). The median follow-up was 31 months (range 24–39 months). Ten patients were randomised prematurely and later failed to meet the inclusion criteria (SCLC, *n*=2; stage IIB–IIIA disease, *n*=5; malignant melanoma, *n*=1; carcinoid, *n*=1; granulomatous disease, *n*=1). Furthermore, two patients were randomised without giving their consent. In total, 432 patients, 218 in the VC and 214 in the GC arm, met the eligibility criteria and constituted the intention-to-treat population for the primary end-point analysis. This accounts for grossly 40% of all stage IIIB–IV NSCLC patients diagnosed in Norway during the study period (personal communication, The Norwegian Cancer Registry). Nine hospitals recruited ⩾20 patients, constituting 70% of the total patient population. The patient flow chart is presented in Figure 1.

Patient characteristics according to treatment groups are given in Table 1. For all patients, median age was 67 years, 20% ⩾75-years old, 61% male, 71% had stage IV disease, 28% PS 2 and 48% adenocarcinoma. The study arms were well balanced with respect to demographic, clinical and histological characteristics.

### Chemotherapy completion

The mean number of chemotherapy courses was 2.7 for the VC and 2.6 for the GC arm. Reasons for treatment termination are given in Figure 1. In the VC arm, 180 patients (83%) received all three cycles, 21 (10%) two cycles, 15 (7%) one cycle and two (1%) no chemotherapy. In the GC arm, the corresponding numbers were 167 (78%), 17 (8%), 26 (12%) and 4 (2%).

Delayed or cancelled vinorelbine or gemcitabine at day 8 due to haematological toxicity was observed in 9.3% (delayed 4.6%; not given 4.8%) of the VC and 18.1% (delayed 10.2%; not given 7.9%) of the GC group (*P*=0.03). Time exceeding 24 days between the main chemotherapy courses occurred in 15% of VC and 23% of GC patients (*P*=0.06).

### Survival

**Figure 2:**
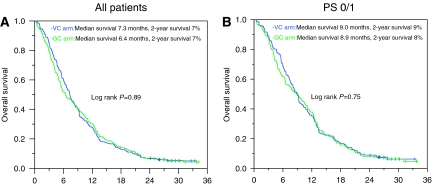
Overall survival according to treatment arms. (**A**) Survival for all study patients; VC (*n*=218, censored *n*=12) and GC (*n*=214, censored=11). (**B**) Survival for PS 0/1 patients; VC (*n*=156, censored *n*=11) and GC (*n*=153, censored *n*=9).

All enrolled patients were included in the survival analyses (*n*=432). There was no difference in overall survival between the treatment arms (*P*=0.89; Figure 2A). The median survival was 7.3 *vs* 6.4 months and the 1- and 2-year survival were 28 and 7% *vs* 30 and 7%, respectively, for the VC and GC arm. Excluding the PS 2 patients, the corresponding median survival was 9.0 *vs* 8.9 months, while the 1- and 2-year survival were 35 and 9% *vs* 38 and 8%, respectively (*P*=0.75; Figure 2B).

The cause of death was recorded in 324 patients. As reported by the local investigators, 282 deaths (87%) were caused by lung cancer. Six deaths were associated with chemotherapy side effects (VC, *n*=2; GC, *n*=4), while the remaining (*n*=36) were classified as other causes.

### QOL

**Figure 3:**
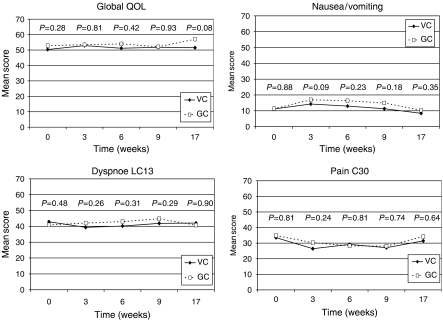
Health-related quality of life scores (weeks 0–17) for global QOL, nausea/vomiting, pain, and dyspnoea according to treatment arm.

The compliance rate with respect to completion of the HRQOL questionnaires was 95 and 98% at baseline and declined to minimum 61 and 60% during the 49-week follow-up for the VC and GC arm, respectively. Mean score analyses were performed for all patients, while only patients with completed baseline HRQOL questionnaires were included in the mean change analyses (VC, *n*=207; GC, *n*=210). The AUC was analysed for patients who had completed all five questionnaires during the period of interest (VC, *n*=111; GC, *n*=97). Mean scores for global QOL, nausea/vomiting, dyspnoea and pain are shown in Figure 3. Mean scores at baseline and weeks 3, 6, 9 and 17 were compared between the treatment arms. The GC arm tended to have slightly worse scores than the VC arm for nausea/vomiting and dyspnoea during therapy, but there were no significant differences for any of the four examined HRQOL items. Neither was there any difference between the VC and GC arm with respect to mean change of scores or AUC from baseline to week 17 (data not shown).

### Toxicity and required interventions

Data on haematological toxicity are presented in Table 2. More grade 3–4 anaemia and thrombocytopenia were observed in the GC arm (*P*<0.01), while there was more grade 3–4 leucopoenia in the VC arm (*P*<0.01). Other treatment side effects and required interventions are given in Table 3. More patients in the GC arm needed blood transfusions (*P*<0.01) or platelets (*P*=0.04), while there was no difference between the arms with respect to neutropenic infections (*P*=0.98). Patients in the GC arm tended to more frequent hospital admissions (*P*=0.10).

### Anticancer treatment beyond the trial regimens

Data on second-line chemotherapy and palliative radiotherapy were available for 95% of the patients and showed no differences between the treatment arms. In the VC *vs* GC arm, 46 *vs* 39% (*P*=0.08), 32 *vs* 25% (*P*=0.23) and 15 *vs* 13% (*P*=0.45) received palliative radiotherapy, second-line chemotherapy, or both.

## DISCUSSION

This randomised trial demonstrates that VC, when compared to GC in NSCLC stage IIIB and IV, is equally effective (median and 2-year survival 7.3 months and 7% *vs* 6.5 months and 7%), but causes significantly less grade 3–4 anaemia and thrombocytopenia.

The main strength of the study is the highly representative patient population. Roughly 40% of all stage IIIB and IV NSCLC patients diagnosed in Norway during the study period were included. Nearly one-third of the included patients had PS 2. Further, the treatment arms were equivalent with respect to chemotherapy delivery schedule, facilitating a direct comparison of the two regimens. The limited number of patients (⩽5) included at each of the smaller hospitals may be a weakness, but constituted only 5% of the study population.

Vinorelbine combined with platinum has proved to be one of the most promising regimens in the adjuvant setting (Douillard *et al*, 2006), and vinorelbin-based therapy is established as a valuable alternative in treatment of advanced NSCLC (Plessen *et al*, 2006). Gemcitabine is more frequently used in palliative treatment of NSCLC. In a meta-analysis including 4556 patients from 13 randomised trials, gemcitabine-platinum doublets were found to be slightly superior to the non-gemcitabine combinations regarding progression-free survival only (Le Chevalier *et al*, 2005). When compared to third-generation platinum-based doublets, the difference was no longer significant (HR 0.93, CI 0.86–1.01).

Whether carboplatin may substitute cisplatin in two-drug platinum-based combinations for advanced NSCLC was investigated by Hotta *et al* (2004) in a meta-analysis including 2945 patients. It was concluded that cisplatin combined with a third-generation agent produced a survival advantage of 11% when compared to carboplatin and the same third-generation agent. The recent CISCA (cisplatin *vs* carboplatin) meta-analysis (Ardizzoni *et al*, 2006), an individual patient data meta-analysis presented at ASCO 2006, found cisplatin-based chemotherapy superior to carboplatin-based with respect to response rate, but not overall survival. However, in palliative treatment of NSCLC, patient QOL, treatment toxicity and time hospitalised are considered more relevant issues.

The median survival in our study is somewhat lower when compared to other phase III trials. For vinorelbine/platinum combinations, recent phase III studies have yielded median survival data ranging from 6.5 to 11 months (Wozniak *et al*, 1998; Kelly *et al*, 2001; Scagliotti *et al*, 2002; Fossella *et al*, 2003; Gebbia *et al*, 2003; Georgoulias *et al*, 2005; Martoni *et al*, 2005; Tan *et al*, 2005; Plessen *et al*, 2006). In the gemcitabine/platinum meta-analysis (Le Chevalier *et al*, 2005), median survival in the gemcitabine group was 9 months, reflecting the mean value for a number of studies (Le Chevalier *et al*, 1994; Cardenal *et al*, 1999; Sandler *et al*, 2000; Scagliotti *et al*, 2002; Schiller *et al*, 2002; Alberola *et al*, 2003; Gebbia *et al*, 2003; Smit *et al*, 2003; Zatloukal *et al*, 2003; Martoni *et al*, 2005; Rudd *et al*, 2005; Sederholm *et al*, 2005). Our shorter median survival is possibly explained by the high proportion of patients with PS 2 (28%), which is the most powerful predictor of survival in NSCLC patients (Stanley, 1980). When PS 2 patients were excluded from our analysis, the median survival was 9.0 *vs* 8.9 months in the VC and GC arm, respectively. Nevertheless, this study shows that overall survival after VC is equivalent to GC in an unselected lung cancer patient population mimicking the everyday clinical setting.

The optimal duration of palliative therapy is debated. The 2003 update of the ASCO guidelines recommended limiting chemotherapy to six cycles in general and stopping treatment after four cycles in stage IV patients who do not respond to treatment. This limitation was based on a British trial comparing three *vs* six courses of mitomycin, vinblastine and cisplatin (Smith *et al*, 2001), and a US study comparing four courses of carboplatin/paclitaxel with the same combination given until progression (Socinski *et al*, 2002). Neither trial showed benefits from longer treatment duration. Additionally, the Norwegian Lung Cancer Study Group recently published a study comparing three *vs* six courses of VC, showing no benefit from the longer treatment (Plessen *et al*, 2006). Consequently, we chose three course regimens for the current study. The selected carboplatin and vinorelbine doses and schedule in the present trial were based on this Norwegian study. The gemcitabine dose resulted from a pilot study and the experience from a phase II study (Kortsik *et al*, 2003). As 83% of the patients in the VC arm and 78% in the GC arm tolerated all three courses, doses and schedules appear appropriate for the study population.

The higher rate of leucopoenia experienced in the VC arm (45 *vs* 30%) did not result in higher infection rates and was mainly laboratory toxicity without direct impact on patients' lives. In contrast, the markedly higher incidence of grade 3–4 anaemia (19 *vs* 6%) and thrombocytopenia (44 *vs* 3%) in the GC arm led to additional symptoms and significantly more frequent transfusions of blood products, requiring hospitalisation and further costs. Haematological toxicity following VC treatment of 159 advanced NSCLC patients was reported by Tan *et al* (2005). In this study, the vinorelbine dose was higher (30 mg m^−2^), and inclusion criteria were more restricted with Karnofsky PS⩾80 and age <75. Leucopoenia was observed less often (22%) and grade 3–4 anaemia significantly more frequent (21%). The latter might be explained by the somewhat higher vinorelbine dose. In the previous Norwegian NSCLC study (Plessen *et al*, 2006), the inclusion criteria were similar and 150 patients received VC chemotherapy doses and schedule identical to the present study. Plessen *et al* (2006) reported slightly less frequent grade 3–4 leucopoenia (35%), whereas the frequencies of grade 3–4 anaemia and thrombocytopenia were similar. In a Swedish phase III study in advanced NSCLC (Sederholm *et al*, 2005), GC treatment yielded the same leucopoenia and thrombocytopenia rates as in our study, but grade 3–4 anaemia was seen in only 5% of the patients. The age distribution was comparable to our study, while the proportion of PS 2 patients was smaller and fewer patients had metastatic disease. Thus, our toxicity data are consistent with previous studies and support the chosen chemotherapy dosage.

The overall compliance rate regarding completion of the HRQOL forms during the study period was 88%, which is equal or better than previous lung cancer studies using the EORTC questionnaire (Cardenal *et al*, 1999; Scagliotti *et al*, 2002; Gridelli *et al*, 2003a, 2003b; Smit *et al*, 2003; Rudd *et al*, 2005; Sederholm *et al*, 2005; Plessen *et al*, 2006). However, the higher frequency of toxicity and interventions after GC therapy were not reflected in any HRQOL difference between the treatment arms. Consistent with the discussion by Scagliotti *et al* (2002), regarding their comparison of gemcitabine-cisplatin and vinorelbine-cisplatin in advanced NSCLC, the timing of HRQOL questionnaire completion at the end of each cycle may mask the effect of acute chemotherapy-induced toxicity.

In conclusion, this randomised comparison of the two platinum-based doublets with vinorelbine or gemcitabine showed equivalent survival and HRQOL, while clinically relevant toxicity was more frequent in the GC arm. To minimise toxicity-related burdens for patients, the VC combination appears an appropriate choice for palliative treatment of advanced NSCLC.

## Figures and Tables

**Table 1:** Patient characteristics at inclusion according to treatment arm

	**VIN/CARBO**		**GEM/CARBO**		
	**(*n*=218)**		**(*n*=214)**		
**Characteristics**	***n***	**%**	***n***	**%**	***P***
*Age* (*years*)					
Median	67		67		0.92
Range	37–86		37–85		
					
*Sex*					
Female	90	41	78	36	0.30
Male	128	59	136	64	
					
*Performance status*					
0/1	156	72	153	71	0.99
2	62	28	61	29	
					
*Extent of disease*					
Stage IIIB	65	30	60	28	0.68
Stage IV	153	70	154	72	
					
*Histology*					
Squamous cell carcinoma	58	26	52	24	0.65
Adenocarcinoma	108	50	101	47	
Large cell carcinoma	11	5	19	9	
Other	41	19	42	20	

**Table 2:** Haematological toxicity according to treatment arm

	**VIN/CARBO**				**GEM/CARBO**				
	***n*=216**				***n*=210**				
	**Grade 3**		**Grade 4**		**Grade 3**		**Grade 4**		
	***n***	**%**	***n***	**%**	***n***	**%**	***n***	**%**	***P***
Anaemia	13	6	0	0	35	16	7	3	<0.01
Leucopoenia	82	38	16	7	57	27	6	3	<0.01
Thrombocytopenia	4	2	1	0.5	53	25	41	19	<0.01

**Table 3:** Side effects and interventions secondary to haematological toxicities according to treatment arm

**Characteristics**	**VC**	**GC**	***P***
*Thrombocytopenic bleeding*			
No. of patients bleeding	1	11	<0.01
Bleeding/patient	0.004	0.05	
Missing (*n*)	3	7	
			
*Neutropenic infections*			
No. of infections	46	48	0.98
Infections/patient	0.21	0.21	
Missing (*n*)	6	8	
			
*Hospital admissions*			
No. of admissions	80	102	0.10
Admission/patient	0.39	0.52	
Missing (*n*)	10	9	
			
*Blood transfusions*			
No. units	136	283	<0.01
Units/patient	0.65	1.36	
Missing (*n*)	2	3	
			
*Platelet transfusions*			
No. of units	5	30	0.02
Units/patient	0.03	0.15	
Missing (*n*)	16	16	

## Appendix

### INVESTIGATORS AT STUDY CENTRES

Ø Furnes, Hammerfest Hospital; PK Skorpen, U Spreng, Stokmarknes Hospital; HH Strøm, P Vanke, Sandessjøen Hospital; M Onkiehong, Rana Hospital; P Reckert, A Boeckmann, Narvik Hospital; A Fossli, B Heermann, L Strauman, Lofoten Hospital; M Jordhøy, A Reigstad, O Alexandersen, TB Nymark, Nordland Hospital Bodø; NG Zafran, Harstad Hospital; E Heitmann, T Børvik, N Ritland, U Aasebø, M Vold, M Petersen, P Røyset, University Hospital of Northern Norway Tromsø; M Heibert, RS Brandsæg, Namsos Hospital; T Naustdal, H Ellekjær, PA Oppegaard, R Kibsgaard, Ø Brenna, H dalen, Levanger Hospital; OF Aasen, I Eskeland, Volda Hospital; B Jakobsen, Molde Hospital; O Herlofsen, Kristiansund Hospital; F Majeed, F Wammer, JÅ Longva, H Fremstad, Erik Liaaen,, Ålesund Hospital; H Hjelde, AS von Krogh, AK Knudsen, S Sundstrøm, E Titova, B Grønberg, T Amundsen, H Hoven, H Tøndel, D Kulosmann, RA Walstad, T Tollåli, M Øyre, A Prestmo, S Sørhaug, S Osen, M Thronæs, H Enger, St Olavs University Hospital Trondheim, A Totland, S Fluge, K Valen, Haugesund Hospital; A Ruud, O Garpestad, M Kosaca, Stavanger University Hospital; CB Alm, Voss Hospital; C von Plessen, Ø Fløtten, H Markova, T Vigander, O Mørkve, A Thelle, A van Lessen, Haukeland University Hospital Bergen; H Rolke, F Gallefoss, G Hoven, Sørlandet Hospital Kristiansand; V Lejlic, E Røyndstrand, Sørlandet hospital Arendal; O Øygarden, Blefjell Hospital Rjukan; Ø Fløtterød, W Gustavsson, Telemark Hospial; OK Andersen, K Semb, Vestfold Hospital Tønsberg; V Stenberg, T Bjørge, H Ranfelt, Ringerike Hospital; Å Skår, Å Hollender, L Rusten, Buskerud Hospital; L Johansen, M Ruppert, Bærum Hospital; A Bailey, A Furuset, V Søyseth, Akershus University Hospital; PF Ekholdt, SØ Gjørstad, Ø Lunde, W Hauge, Fredrikstad Hospital; P Brunsvig, Å Brattland, R Hatlevoll, H Stensheim, Radium Hospital Oslo; A Fjell, B Bergholtz, Aker University Hospital; F Stornes, O reitan, M Wang, K Hornslien, L Breidablikk, Ullevål University Hospital.
